# A Western Diet High in Phosphate Primes the Development of the CKD-Mineral Bone Disorder in an Alport Syndrome Model

**DOI:** 10.34067/KID.0000001065

**Published:** 2026-01-02

**Authors:** Matthew J. Williams, Hiral M. Patel, Carley B. Halling, Brian N. Finck, Keith A. Hruska

**Affiliations:** 1 Department of Pediatrics, Nephrology, Washington University in Saint Louis, Saint Louis, Missouri; 2 Department of Medicine, Nutritional Science and Obesity Medicine, Washington University in Saint Louis, Saint Louis, Missouri; 3 Department of Cell Biology, Washington University in Saint Louis, Saint Louis, Missouri; 4 Department of Medicine, Nephrology, Washington University in Saint Louis, Saint Louis, Missouri

**Keywords:** cardiovascular disease, CKD, hyperphosphatemia, mineral metabolism, mitochondria

## Abstract

**Key Points:**

- An animal protein high-phosphate diet decreased cardiac mitochondrial oxidative phosphorylation in mice with normal kidney function.
- The animal-based protein high-phosphate diet worsened the severity of several CKD-mineral bone disease components in CKD.
- The effects of the diet during normal renal function prime the development and severity of the CKD-mineral bone disease during kidney disease progression.

**Background:**

CKD-mineral bone disorder (CKD-MBD) is a syndrome that contributes to cardiovascular mortality. We have shown that CKD decreases cardiac mitochondrial function independent of vascular disease and before cardiac hypertrophy. Hyperphosphatemia, a component of the CKD-MBD occurring in later stages of CKD, has been shown to stimulate vascular calcification (VC). In a mouse model of Alport syndrome CKD resistant to VC, we examine the effects of a high-phosphate Western-type diet (HP) on the components of the CKD-MBD, including cardiac respiration.

**Methods:**

*Col4a5*-deficient mice and wild-type (WT) littermates were fed an animal protein 1.2% high-phosphate diet or a standard vegetable protein 0.6% phosphate diet. At CKD progression equivalent to human CKD stages 4–5, we examined cardiac tissue for mitochondrial respiration, kidney histology for fibrosis, blood for BUN and CKD-MBD components, kidney tissue for klotho production, and aorta for VC.

**Results:**

The HP diet produced hyperphosphatemia in the CKD animals compared with WT. The diet increased plasma parathyroid hormone (PTH; 17-fold), fibroblast growth factor 23 (FGF23) intact (14-fold), and reduced kidney klotho mRNA and protein more than 50%. Alport CKD mice fed the HP diet showed a reduction in cardiac mitochondrial complex 2–mediated oxidative phosphorylation, and higher levels of plasma PTH and FGF23 than CKD mice fed the vegetable protein diet. Comparing WT groups, the HP diet increased PTH and intact FGF23, reduced renal klotho, and decreased cardiac mitochondrial oxidative phosphorylation capacity. VC was not induced by the HP diet.

**Conclusions:**

The Western-style high-phosphate diet primed the development of the CKD-MBD in nondiseased animals and worsened the CKD-MBD during CKD progression. Cardiac respiration, renal klotho, FGF23, and PTH are affected by a high-phosphate diet even with normal kidney function, suggesting a need for early intervention in the management of phosphate homeostasis as a component of CKD therapy.

## Visual Abstract

**Figure:**
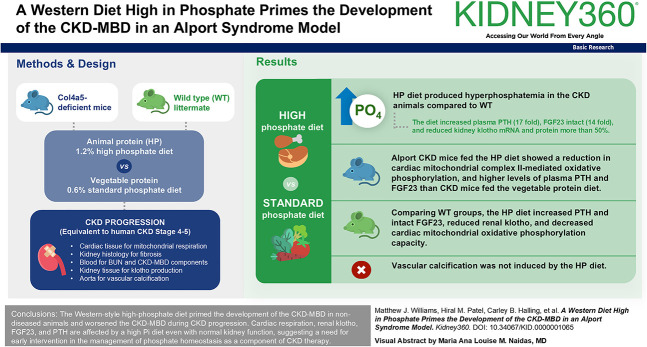

## Introduction

CKD is a public health pandemic affecting approximately 850 million people including 5.5 million Americans.^1^ Advanced CKD is associated with several adverse clinical outcomes, such as accelerated cardiovascular (CV) diseases, kidney failure requiring KRT, mineral bone disease, anemia, metabolic and endocrine abnormalities, and poor quality of life.^2,3^ Patients with CKD stage 3 and 4 have a two-fold to three-fold increased risk of CV mortality, and this risk is further increased in patients with CKD stage 5 on dialysis.^4,5^ The CV mortality associated with CKD is remarkable in that the excess risk compared with the general population is not due to established CV disease risk factors.^6^ Rather, studies over the past two-plus decades have identified nontraditional risk factors as contributing to CKD-associated mortality. These nontraditional risk factors include hyperphosphatemia,^7^ fibroblast growth factor 23 (FGF23),^8,9^ *α*klotho deficiency,^10–13^ CKD-stimulated vascular disease,^14–16^ and renal osteodystrophy, now referred to as CKD-associated osteoporosis.^17^ These risk factors are components of the syndrome named the CKD-MBD (CKD-mineral bone disorder)^18,19^ in recognition of their role in CV complications of CKD.^5,20–22^ Shortly after the CKD-MBD designation, it was found to be a uniform result of kidney diseases and to begin early in the course of disease (stage 2 CKD).^23–26^ At these early stages, the CKD-MBD consists of vascular disease (especially arterial stiffness), osteodystrophy, elevated FGF23, and decreased *α*klotho.^12,23,26,27^ As kidney disease progresses, the familiar clinical elements of the CKD-MBD become manifest, and calcitriol deficiency, hyperparathyroidism, hyperphosphatemia, hypocalcemia, and cardiac hypertrophy become prevalent.^28–30^

Hyperphosphatemia, a component of the CKD-MBD, is associated with all-cause and CV mortality in CKD^7,31,32^ and in the general population.^33^ As early as 1982, control of inorganic phosphate (Pi) has been considered as a potential therapeutic option in CKD progression.^34^ Hu *et al*.^10^ challenged heterozygote klotho deficient (*kl*/*+*), wild type (WT), and klotho transgenic overexpressing (*Tg-Kl*) mice at 6 months of age with a 2% high-phosphate (HP) diet for 12 weeks. They showed that cardiac hypertrophy and fibrosis were exaggerated in the *kl*/*+* mice and lessened in *Tg-Kl* mice compared with WT mice and that the high-Pi diet induced hypertrophy and fibrosis in the WT mice.

Faul *et al*.^35^ showed that FGF23 signaling independent of klotho induces cardiac hypertrophy and contributes to the cardiac fibrosis in CKD. We^36^ have shown that CKD decreases cardiac mitochondrial function before cardiac hypertrophy. Hu *et al*. showed that the cardiac FGF23 effect in CKD is dependent on low klotho levels.^10^ Furthermore, klotho protein in kidney, plasma, and urine was decreased by phosphate loading, and the high plasma Pi and FGF23 levels were exaggerated in *kl*/*+* mice and attenuated in *Tg-Kl* mice.^10,37^ Yanucil *et al*.^37^ showed that the pathologic cardiac actions of FGF23 are modulated by klotho and heparin. Recently, Fuchs *et al*.^38^ showed that FGF23 and fibroblast growth factor receptor 4 promote cardiac remodeling and affect cardiac respiration through increased glycolysis in CKD.

We have developed a murine model of human CKD, *Col4a5* deficiency Alport syndrome on the C57Bl6J background. This model does not express conduit artery disease at CKD equivalency to human stages 4–5 CKD, and we have reported decreased cardiac mitochondrial respiration, showing effects of CKD on the heart independent of vascular disease and before left ventricular hypertrophy.^36^ Here, we have found that a Western diet high in phosphate worsens the decrease in cardiac mitochondrial respiration in the Alport mice with CKD equivalent to human stages 4–5 CKD and that diet effects on cardiac respiration were found even in mice with normal kidney function. In fact, the HP Western style diet affected multiple components of the CKD-MBD in wild-type mice and worsened the severity of the CKD-MBD in CKD. The Western HP diet decreased cardiac mitochondrial complex one leak respiration and complex 2 mediated oxidative phosphorylation (OXPHOS) in WT mice. This primed the development of the CKD-MBD during CKD progression. Finally, we found that hyperphosphatemia was not sufficient to induce vascular calcification (VC) or vascular smooth muscle cell (VSMC) transdifferentiation in the Alport CKD mice.

## Methods

### Groups and Statistics

Four experimental groups are evaluated in this study: Alport CKD mice fed a high-Pi Western-type diet (CKD HP), their wild type littermates (WT HP), and Alport CKD mice fed a standard vegetable-based mouse diet and their WT littermates (WT). Comparisons were made between WT and CKD groups of each diet to show effects of CKD and between WT animals of both diets and between CKD animals of both diets to show effects of Western high-phosphate diet in WT animals and in CKD animals. The technicians responsible for animal euthanasia and data collection were blinded to animal genotype and diet. Samples were selected for biochemical assays and histologic analysis based on similar euthanasia dates, availability, and to equalize division among groups where possible. The technicians performing the assays and quantitation were blinded to the sample groups, and the order of processing of samples was determined by random number generation.

**Figure 1:**
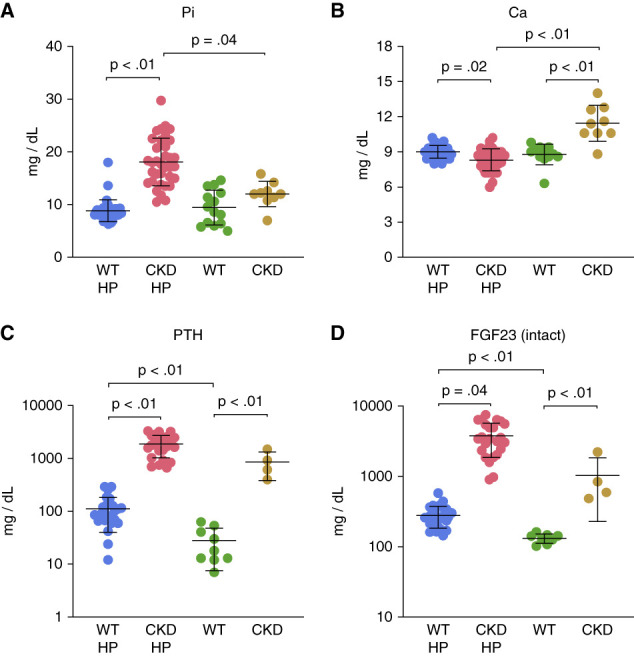
**Serum chemistries and plasma PTH and FGF23 in WT and CKD mice fed either the Western-style high-phosphate diet (WT HP and CKD HP) or vegetable protein–based standard chow diet (WT and CKD).** The data show that CKD produced the CKD-MBD in both diet groups, but it was exacerbated with HP Western diet. (A) Serum Pi is significantly increased in CKD with mice on high-phosphate Western diet. (B) Serum Ca levels were decreased in CKD mice on a high-phosphate diet due to the hyperphosphatemia. Serum Ca levels were increased in CKD mice on the vegetable protein diet with low phosphate availability due to the increase of free Ca from gastrointestinal absorption and the effects of hyperparathyroidism on bone resorption. (A and B) Western diet groups *n*=35–42; vegetable diet groups, *n*=11–16. Plasma levels of PTH (C) and intact FGF23 (D) are significantly elevated in CKD mice on either the high-phosphate diet or standard chow diet compared with their WT littermates on the same diet. PTH (C) and intact FGF23 (D) levels also show increase in WT mice fed high-phosphate diet compared with standard chow-fed mice. (C and D) Western diet: WT HP, *n*=28; CKD HP, *n*=24. Vegetable diet WT, *n*=9; CKD, *n*=4. Significance differences between groups were determined using Kruskal-Wallis H test with Bonferroni correction for multiple pairwise comparisons. Data displayed on log10 scale (C and D). Data are represented as mean±SD. Includes data limited by animals with associated BUN <35 for WT and >43 for CKD. Ca, calcium; CKD-MBD, CKD-mineral bone disorder; HP, high-phosphate; FGF23, fibroblast growth factor 23; Pi, inorganic phosphate; PTH, parathyroid hormone; WT, wild type.

All data groups were examined with Shapiro–Wilk test for normality and Levene test for equality of variance across groups. Significant differences between two groups with normal distribution and equal variance between groups was determined with Student's *t* test. Significance differences between datasets with more than two groups, approximate normal distributions, and equal variance across groups were determined by ANOVA with the Tukey multiple comparison test. Significance differences between datasets with more than two groups, approximate normal distributions, and unequal variance across groups were determined by Welch ANOVA with Dunnett T3 multiple comparison test. Otherwise, significance was determined using Kruskal–Wallis H test with Bonferroni correction for multiple pairwise comparisons. Rare extreme outliers as identified using the interquartile range method in SPSS were removed from calculations for significant differences. All quantitative data are presented as mean±SD, and *P* < 0.05 was considered statistically significant. Significance bars are only shown if *P* < 0.05. Analyses of plasma protein levels (Figure 1, C and D) were performed on log10-transformed data. All data analyses were performed using IBM SPSS Statistics 29.

### Alport Mice

We used the murine homolog of X-linked Alport syndrome, which is a deficiency in the gene for the *α*5 chain of type 4 collagen, *Col4a5*, as a model of human CKD.^39^ WT males were bred with *Col4a5*^+/−^ females to generate males with (CKD) and without (WT littermates, WT) *Col4a5* knockout on a mixed C57BL/6J background. *Col4a5*^+/−^ female selection was based on production of male progeny without VC. Hemizygote males developed spontaneous kidney disease and were used to study the CKD-MBD. Mice received either a Western (casein-based) 1.2% high-Pi, 0.6% Ca diet (Dyets, Inc., 113321), or a standard (vegetable protein-based) chow diet (LabDiet 5053—PicoLab Rodent diet 20, 0.61% total Pi with 0.33% non–phytate-bound, 0.8% Ca) beginning at 75 days old (do) until euthanasia. Protein content between the two diets was the same. Euthanasia date for standard diet-fed mice was designed for 225 days of life, but mortality was high in mice with CKD on this diet. Expecting increased mortality with CKD mice fed Western HP diet, the planned euthanasia date for mice on this diet was shortened to 190 days to conserve animals. The Institutional Animal Care and Use Committee at Washington University in Saint Louis approved all animal studies (protocol ID 24-0117), and they adhere to the National Institutes of Health Guide for the Care and Use of Laboratory Animals.

### Tissue Collection

Euthanasia of mice was performed under injection anesthesia (xylazine 13 mg/kg body weight and ketamine 87 mg/kg body weight) or inhalant anesthesia (2% isoflurane). Blood was taken by intracardiac stab, and the kidneys, hearts, and aortas dissected *en bloc*. Blood samples were processed with BD Microtainer gold serum separator tubes for serum and lavender K2EDTA tubes for plasma. All blood samples were placed on ice at collection and processed samples were stored at −80°C or below until use.

### Serum and Plasma Assays

Serum was analyzed for BUN, serum calcium, and serum phosphate by Midwest Vet Labs (St. Louis, MO) using a Beckman Coulter AU480 Chemistry Analyzer. Plasma protein levels of intact parathyroid hormone (PTH; 60–2305, Quidel Corporation), *C*-terminal FGF23 (60–6300, Quidel Corporation), and intact FGF23 (60–6800, Quidel Corporation) were determined by ELISA.

### Kidney Fibrosis and Cellularity

Picrosirius red staining was used to determine progression of interstitial fibrosis. Kidney tissues were fixed in 10% neutral buffered formalin overnight, then transferred to 70% ethanol at 4°C, and embedded in paraffin. Embedded tissues were sectioned on the midline frontal plane to 5 micron thickness and stained using the Picrosirius red method. Slides were deparaffinized in xylene and rehydrated in a graded ethanol series. Nuclei were stained with Wiegert hematoxylin for 8 minutes, followed by slide wash in tap water for 10 minutes. Slides were then stained with 0.1% (w/v) Direct Red 80 (Millipore Sigma, 365548) in saturated aqueous picric acid (Millipore Sigma, P6744) for 1 hour followed with two washes in 0.5% (v/v) aqueous acetic acid. Finally, the slides were dehydrated with three 1-minute washes in 100% ethanol before clearing in xylene and mounting. All histology processing and staining was performed by the Musculoskeletal Research Center Histology Core at Washington University in St. Louis.

Quantitation of fibrosis was determined using fraction of red-stained area in total renal parenchyma. Whole tissue sections were imaged under bright field illumination at 20× in same session at the Hope Center Alafi Neuroimaging Lab using a Hamamatsu NanoZoomer 2.0-HT System with NDP.scan 2.5 software. Images were scaled, converted to 24-bit RGB, and processed using ImageJ FIJI distribution.^40^ Regions of interest were then scribed to include total area of the tissue. Area fraction of positive staining was calculated using color thresholding plug-in and the hue, saturation, and brightness color model. Parameters were set to isolate red-stained area that increases in fibrosis. Quantitation of cellularity was determined using a similar method. In this case, color determination was set to capture dark blue–stained cell nuclei.

### RNA Preparation and Real-Time PCR

Total RNA from tissue was isolated and purified using TRIzol (15596018, ThermoFisher Scientific) with tissue disruption by the TissueLyser II (Qiagen), Phasemaker tubes (A33248, ThermoFisher Scientific), and PureLink RNA Mini Kit (12183018a, ThermoFisher Scientific) with PureLink DNase (12185010, ThermoFisher Scientific) according to manufacturers' instructions. RNA concentration and quality was determined using the NanoDrop OneC spectrophotometer (ThermoFisher Scientific). cDNA was generated for real-time PCR (qPCR) using the High-Capacity cDNA Reverse Transcription Kit (4368814, ThermoFisher Scientific). Quantitative PCR analysis was performed with the StepOnePlus Real-Time PCR System (Applied Biosystems) using the PowerUp SYBR Green Master Mix (A25742, Applied Biosystems) run in fast cycling mode. Expression levels were normalized by standardizing cDNA concentration. All biologic samples were run using two technical replicates. All primers for qPCR were purchased from the LabReady Oligo Service from Integrated DNA Technologies, Inc. (Coralville, Iowa). The list of primers is presented in Table 1.

**Table 1:** Quantitative PCR primers

Target Gene (Mouse)	Symbol	Forward Primer (5ʹ–>3ʹ)	Reverse Primer (5ʹ–>3ʹ)
Hypoxanthine phosphoribosyltransferase 1	*Hprt1*	ATGCCGAGGATTTGGAAAAAGTGT	GTGATGGCCTCCCATCTCCTT
Klotho	*Kl*	TTC​CCT​GTG​ACT​TTG​CTT​GGG	TCC​CAC​AGA​TAG​ACA​TTC​GGG​TC
Runt related transcription factor 2	*Runx2*	GTG​GCC​ACT​TAC​CAC​AGA​GC	GGG​ATG​AGG​AAT​GCG​CCC​TA
Sclerostin	*Sost*	GAC​CTC​CCC​ACC​ATC​CCT​AT	TGT​CAG​GAA​GCG​GGT​GTA​GTG
Transgelin	*Tagln*	GGC​CTT​TAA​ACC​CCT​CAC​CCA​G	TTG​TTG​GCC​ATG​TTG​AGG​CAG

### Aortic Calcification

Calcium phosphate in aortas was visualized with Von Kossa histology staining. Freshly excised aortas were cleaned of perivascular adipose tissue, flash frozen in liquid nitrogen, and stored at −80°C. Once all the frozen aortas in the experimental groups were collected, they were briefly thawed at 4°C, straightened, immersed in cold (on ice) 10% formalin, fixed overnight at 4°C, transferred to 70% ethanol, and embedded in paraffin. Embedded aortas, oriented to capture the full length of the tissue, were sectioned twice at 5-micron thickness, at 50-micron difference in depth. Following deparaffinization and hydration, Von Kossa's stain method progressed with exposure to 5% silver solution (Millipore Sigma, 209139) under bright light for 1 hour, four rinses with distilled water, 5-minute exposure to 5% sodium thiosulphate, tap water wash with distilled water rinse, counterstain with Nuclear Fast Red (Millipore Sigma, N3020) for 5 minutes, wash in tap water, slide dehydration in a graded ethanol series, clear, and coverslip. All histology processing and staining was performed by the Musculoskeletal Research Center Histology Core at Washington University in St. Louis. Whole aorta sections were imaged under bright field illumination at 20× in same session at the Hope Center Alafi Neuroimaging Lab using a Hamamatsu NanoZoomer 2.0-HT System with NDP.scan 2.5 software. Scanned slides were examined by technician using NDP.view 2.7.52.

### Protein Extraction and Immunoblot Analysis

Full length frozen aortas were homogenized in ice-cold RIPA Lysis and Extraction Buffer (ThermoFisher Scientific, 89900) with Protease Inhibitor Cocktail (Millipore Sigma, P8340) and PhosSTOP phosphatase inhibitor (Millipore Sigma, 4906845001) using a TissueLyser II (Qiagen). Total protein of each sample lysate was determined with the Pierce BCA Protein Assay Kit (ThermoFisher Scientific). Lysates were then prepared under reducing conditions and loaded with equivalent total protein into a 10% TGX precast protein gel (Bio-Rad) for protein separation using gel electrophoresis. Proteins in the finished gel were transferred to a 0.45 polyvinylidene fluoride membrane using a Trans-Blot Turbo (Bio-Rad) according to manufacturer's guidelines. Resulting blots were blocked with 1% milk (Bio-Rad, 1706404) or Li-Cor Intercept Blocking Buffer (927-600001) in tris-buffered saline and probed with antibodies to 1:1000 RUNX2 (D1L7F) (Cell Signaling Technology Cat# 12556, RRID:AB_2732805) or 1:500 Klotho (KM2076; Cosmo Bio Cat# KAL-KO603, RRID:AB_3390355) in addition to housekeeping control 1:500 *β*-actin (C4) Alexa Fluor 680 (Santa Cruz Biotechnology Cat# sc-47778 AF680, RRID:AB_626632). IRDye 800CW donkey anti-rabbit (LI-COR Biosciences Cat# 926-32213, RRID:AB_621848) and IRDye 800CW goat anti-rat (LI-COR Biosciences Cat# 926-32219, RRID:AB_1850025) at 1:5000 were used as secondary antibodies. Near-infrared fluorescence imaging of multiplexed blots was conducted by the Bio-Rad ChemiDoc MP, and quantitative analysis was performed using Bio-Rad Image Lab 6.1.

### Mitochondrial Respiration

**Figure 2:**
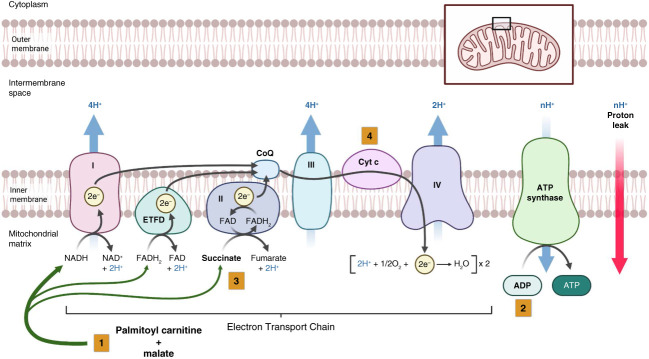
**Measurement of mitochondrial respiration using the Oroboros Oxygraph-2K.** Step 1: addition of PC+M to permeabilized cardiac fibers. This supplies fatty acid that is metabolized with *β*-oxidation and citric acid cycle to provide electron donors NADH and FADH_2_ to the ETC. A majority of the electron flow is facilitated through CI, with minor contributions due to *β*-oxidation from ETFs through ETFD and succinate through CII. *β*-oxidation also produces acetyl-CoA, which stimulates production of succinate through the citric acid cycle cycle. The electron flow from CI, ETFD, and CII moves through CoQ, CIII, Cyt c, and then to complex IV, resulting in reduction of oxygen. This ETC generates a proton gradient in the intermembrane space through activation of the proton pumps CI, CII, and complex IV. This proton gradient produces a proton motive force that is used to activate ATP synthesis in the presence of ADP. In addition, this proton motive force drives protons across the inner mitochondrial membrane, bypassing ATP synthase (Proton leak). The addition of PC+M without ADP maximizes the proton gradient, and low-level oxygen consumption is stimulated by leak of protons across the inner mitochondrial membrane. This Leak Respiration is primarily mediated by CI. Step 2: sequential addition of ADP to cardiac fibers activates ATP synthase (CV), which leverages the proton gradient to produce ATP. This is considered experimental state 3 respiration, and in this case, it represents maximum OXPHOS driven by fatty-acids and principally controlled by CI (CI OXPHOS). Step 3: addition of a high concentration of succinate (S) stimulates contribution of CII and pushes the cardiac fibers to maximum OXPHOS Capacity, also state 3 respiration. Step 4: cytochrome C is added to check for differences in mitochondrial membrane integrity between groups. Created in BioRender. Williams, M. (2025) https://BioRender.com/s21h7nj. CI, complex I; CII, complex II; CoQ, coenzyme Q; CV, cardiovascular; Cyt c, cytochrome c; ETC, electron transport chain; ETF, electron-transferring-flavoproteins; ETFD, ETF dehydrogenase; OXPHOS, oxidative phosphorylation; PC+M, palmitoyl carnitine and malate.

High-resolution respirometry was performed, as previously described^36^ at the Cellular and Molecular Biology Core of the Washington University Nutrition Obesity Research Center, using the methods of Ferey *et al*. and Ojuka *et al*.^41,42^ for mitochondrial physiology.^36^ A freshly excised 2–3 mg section of the left cardiac ventricular lateral wall was placed in biopsy preservation buffer, permeabilized with saponin, transferred to MirO5 mitochondrial respiration medium, blotted dry, and then weighed. Prepared tissue was placed in an Oxygraph-2K (OROBOROS Instruments, Innsbruck, Austria) chamber containing 2 ml of MirO5 mitochondrial respiration medium, 20 mM creatinine monohydrate, and 10 *μ*M blebbistatin. To measure O_2_ flux, the following substrates were added sequentially (Oroboros Instruments; final concentrations indicated): 0.5 mM malate and 25 mM palmitoyl carnitine; 5 mM ADP; 10 mM succinate; and 4 mM cytochrome C, and measurements were performed at steady state with DatLab Software. The protocol is similar to that recently used for skeletal muscle in diabetic CKD.^43^ Figure 2 presents the effects of these substrates on the mitochondrial OXPHOS metabolic pathway. The technician performing respirometry was blinded to the sample groups.

## Results

**Figure 3:**
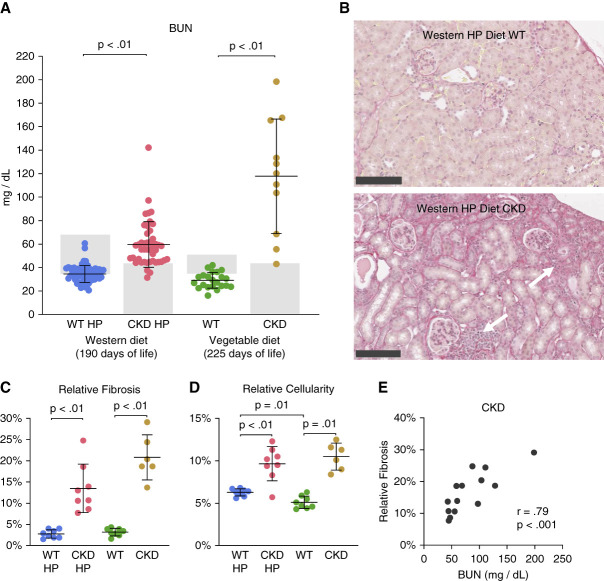
**BUN levels and renal fibrosis in the Alport mice.** The four groups of mice include 190 day old Alport CKD mice and WT littermates fed Western-type high-Pi animal-based protein diet (CKD HP and WT HP) and 225 days old Alport CKD and WT mice fed standard vegetable-based diet. (A) BUN levels were elevated in Alport CKD mice fed either Western-type high-phosphate (Pi) diet or standard vegetable-based protein diet compared with their wild-type littermates. BUN levels did not differ significantly in the WT mice between the two diets. Western diet: WT HP, *n*=62; CKD HP, *n*=45. Vegetable diet WT, *n*=22; CKD, *n*=11. Shaded areas indicate study animals excluded from Figures 1, 4 and 5 for elevated BUN (>35) in WT groups or lack of disease development in CKD groups (BUN <43). Data are represented as means±SD. Significant differences between groups were determined without exclusions using Kruskal-Wallis H test with Bonferroni correction for multiple pairwise comparisons. (B) Representative picrosirius red-stained kidney sections from 190 days old Alport CKD mice fed the Western-type high-Pi diet and WT control. Areas of increased interstitial cellularity are indicated with white arrows. Bar is 250 microns. (C) Quantitation of renal fibrosis calculated by total area fraction of red stain in whole tissue images. Fibrosis was markedly increased in the CKD groups compared with WT. (D) Quantitation of interstitial cellularity by area fraction of nuclear stain was increased in the CKD groups compared with WT. The cellularity did differ between the two WT groups (*P* = 0.01), but the effect was minor. (C and D) Group sizes were *n*=6–8 for fibrosis and cellularity scoring. Data are represented as means±SD. Significant differences between groups were determined using Welch ANOVA with Dunnett T3 multiple comparison test. (E) There was a strong, positive relationship between BUN and renal fibrosis measurement in all CKD mice as indicated by Pearson correlation coefficient (*r*[12]=0.79, *P* < 0.001).

Alport CKD mice in the standard vegetable diet group experienced higher than expected mortality (43%) before planned euthanasia date of 225 days old (Table 2). We expected a further increase of mortality with mice fed Western animal protein-based 1.2% HP diet. Therefore, to conserve animals, we reduced euthanasia date for this group to 190 days old. At this end point, the CKD mice fed a Western-type diet had a 5% mortality (Table 2). Figure 3A shows that the serum BUN levels were significantly increased in Alport CKD compared with WT littermates for the 190 day old mice fed the Western high-Pi diet and the 225 day old mice fed the standard vegetable-based diet. Kidney pathology showed a severe fibrosis and cellular infiltration from the tubulointerstitial nephritis in the 190 day old Western high Pi diet-fed CKD animals (Figure 3B). Tubulointerstitial nephritis ending in fibrosis is the pathologic hallmark of Alport syndrome kidney disease.^44^ Fibrosis and cellular infiltration was also severe in the 225-day-old CKD mice fed the vegetable-based diet (Figure 3, C and D). Figure 3E shows that the measurement of relative fibrosis is strongly correlated with BUN measurements in Alport CKD mice. In the CKD animals fed the vegetable-based diet, the mean BUN and relative fibrosis level trended higher than the animals fed the Western high-Pi diet. This can be explained by the increased age of that group.

**Table 2:** Improved mortality in experimental Alport CKD mice with reduced euthanasia end point

Diet	Group	Days of Life	Mortality
Standard chow	WT	225	0% (0/25)
Standard chow	CKD	225	43% (12/28)
Western high-phosphate	WT HP	190	2% (2/116)
Western high-phosphate	CKD HP	190	5% (8/159)

Mortality includes mice that have died before targeted euthanasia due to disease or were euthanized before specified euthanasia date due to severe disease. Mice that died due to nondisease causes were not included in total. CKD: Alport *Col4a5*-, standard chow diet: vegetable-based protein (0.6% phosphate); CKD HP: Alport *Col4a5*-, Western high-phosphate diet: animal-based protein (1.2% phosphate), WT HP and WT: wild type littermates to CKD mice. HP, high-phosphate; WT, wild type.

Alport syndrome, and as represented in this model, is variable in disease progression. Some *Col4a5*-deficient animals did not show significant elevations of BUN and fibrosis to represent advanced disease, more so in the younger 190 day CKD HP group (Figure 3A). In addition, some WT animals showed an increase in BUN, suggesting early kidney dysfunction. The objective of this study is to examine the contribution of Western type HP diet on the CKD-MBD, including cardiac respiration, and not on the progression of Alport CKD. Therefore, the data in the following figures as indicated were selected from animals with BUN >43 for Alport CKD, or BUN <35 for WT littermates. These bounds prevented inclusion of WT animals that showed decreased kidney function, removed animals from the *Col4a5*-CKD groups that showed poor disease progression, and minimized the total number of animals excluded in the dataset. The normal range of BUN in C57Bl6J older male mice is 21.8–32.3 mg/dl, encompassing 95% of data in Mazzaccara *et al*. (2008).^(45)^ Inclusion criteria based on BUN serves to dampen the effects of comparing animals at two different age groups in both WT and CKD animals; however, one interpretation of the proceeding results is that the Western high-Pi fed mice are younger with less advanced CKD.

The Western-type high-Pi diet produced hyperphosphatemia and hypocalcemia in the Alport CKD animals (Figure 1, A and B). Plasma levels of the CKD-MBD components, PTH and FGF23, are shown in Figure 1, C and D. PTH levels were significantly increased in the 190-day-old high Pi-fed Alport CKD mice to 1880 pg/ml from 110 pg/ml in the WT mice (Figure 1C). In 225-day-old normal Pi-fed mice, PTH levels in the Alport CKD mice were 850 pg/ml compared with 30 pg/ml in the WT littermates. The high-Pi diet even increased PTH levels in the WT littermates when referenced against the normal Pi-fed WT mice. FGF23 intact hormone levels were increased to 3780 pg/ml in Alport CKD mice from 280 pg/ml in WT mice fed the Western high-Pi diet (Figure 1D). Intact FGF23 hormone levels in the normal Pi-fed Alport CKD mice were 640 pg/ml compared with 120 pg/ml in the WT littermates (Figure 1D). *C*-terminal FGF23 protein levels show similar trends between groups (Supplemental Figure 1). The Western high-Pi diet worsened the FGF23 arena in the CKD mice and even increased FGF23 levels in the WT littermates referenced against the normal Pi-fed WT mice.

**Figure 4:**
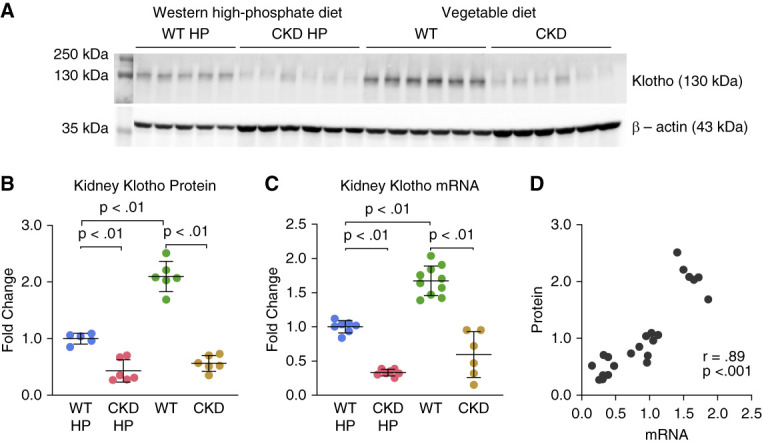
**Klotho expression is reduced in Alport CKD mice and in WT mice fed a Western high-Pi diet.** (A) Kidney klotho protein immunoblot of WT and CKD mice fed either high-phosphate Western diet (HP) or vegetable-based standard diet (*n*=5–6) with relative quantification. (B) Significant differences between groups were determined using ANOVA with Tukey multiple comparison test. (C) Relative kidney klotho mRNA levels (*n*=6–10). Klotho mRNA expression was normalized to *Hprt1*. Significant differences between groups were determined using Welch ANOVA with Dunnett T3 multiple comparison test. All mRNA samples were derived from the same kidneys used in klotho protein analysis (A). (D) Correlation between mRNA and protein klotho levels is strong and positive as indicated by Pearson correlation coefficient (*r*[21]=0.89, *P* < 0.001). Data are represented as means±SD. All data limited by animals with associated BUN <35 for WT and >43 for CKD. See Supplemental Figure 3 for uncut blot.

Kidney αklotho levels in Alport mice fed the Western high-Pi diet were reduced compared with WT (Figure 4A). Klotho was reduced to similar levels in 225 do Alport CKD mice fed regular chow (Figure 4A). However, comparison of *α*klotho levels between the WT groups (Figure 4B) revealed that the high-Pi Western diet reduced renal klotho levels in WT mice. This effect was confirmed with tissue mRNA analysis (Figure 4C), and correlation between mRNA and protein measurements was strong (Figure 4D). Soluble circulating Klotho (sklotho) levels were not determined, due to the lack of validation and comparability in various murine ELISAs. We did not possess the serum stock to independently validate Klotho ELISAs.

### Cardiac Respirometry

**Figure 5:**
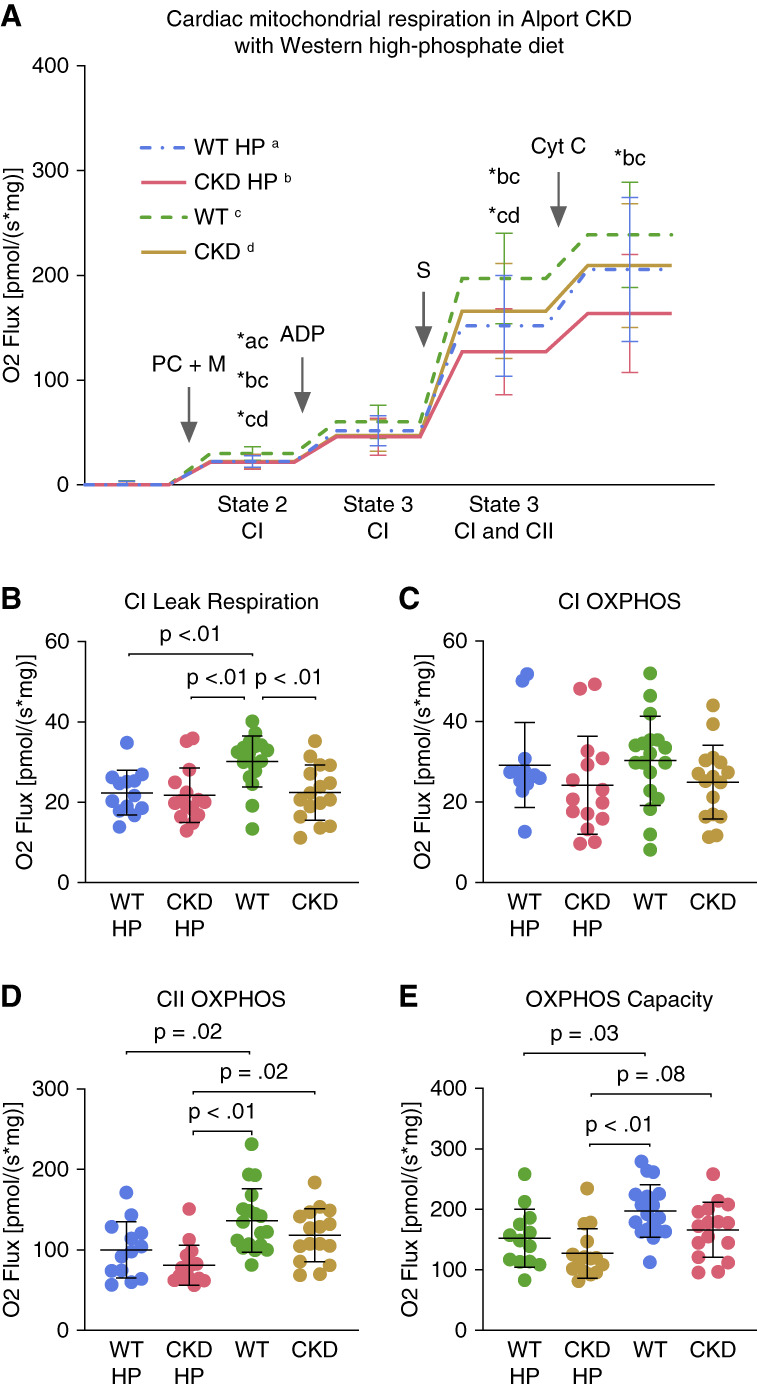
**Cardiac mitochondrial function is reduced in Alport CKD mice fed Western HP diet at 225 days of life.** Groups include CKD and WT littermates (WT) fed HP diet and CKD and WT animals fed standard diet (*n*=13–18). (A) Oxygen consumption rate of isolated cardiac fibers in OROBOROS Oxygraph-2k chamber in response to addition of substrates as described in Figure 2 to stimulate the ETC and ATP production. PC+M, addition of palmitoyl carnitine and malate to permeabilized cardiac fibers to measure leak respiration mediated by CI (B: CI Leak Respiration); ADP, sequential addition of ADP to measure fatty acid–driven OXPHOS (state 3 respiration) capacity mediated by CI (C: CI OXPHOS); S, addition of succinate (S) to measure maximum OXPHOS (E: OXPHOS Capacity) and derive OXPHOS mediated through CII complex (D: CII OXPHOS), also state 3 respiration; Cyt C is added to check for differences in mitochondrial membrane integrity between groups. The HP Western diet significantly reduces OXPHOS capacity in both WT and CKD animals compared with WT animals on normal diet. Significant differences between groups were determined using ANOVA with Tukey multiple comparison test. Data are represented as means±SD. Includes data limited by animals with associated BUN <35 for WT and >43 for CKD.

High-resolution respirometry was used to determine mitochondrial function in mouse cardiac tissue bundles. For this cohort, data were collected at 225 days of life for all groups. Figure 5A shows the experimental protocol and results. Figure 5B shows that oxygen consumption in fatty-acid driven leak respiration is suppressed in Alport CKD animals, and WT and Alport CKD animals fed a Western HP diet, compared with WT animals fed standard diet. In this state, ADP is not available to allow ATP synthase to generate ATP, and the proton gradient is increased to a maximum due to activation of the electron transport chain by palmitoyl carnitine and malate. The elevated proton gradient stimulates proton leak or return of protons to the mitochondrial matrix without phosphorylation; this process allows continued activation of the electron transport chain and low-level oxygen reduction. In Figure 5C, the addition of ADP substrate allowed OXPHOS primarily mediated through complex I (state 3 respiration). Groups were not significantly different. However, Figure 5D shows that complex II–mediated OXPHOS (determined after addition of succinate) revealed significant drops in O_2_ Flux in both WT and CKD animals fed Western HP diet compared with their counterparts fed standard diet. Both complex I-mediated state 2 respiration (Figure 5B) and complex II-mediated OXPHOS (Figure 5D) contributed to group differences observed in OXPHOS capacity (Figure 5, A and E). WT animals fed a HP diet had significantly reduced mitochondrial OXPHOS capacity compared with WT animals fed a standard diet and Alport CKD animals fed a Western HP diet trended to a reduced oxygen flux compared with standard diet-fed animals.

We have previously shown that cardiac hypertrophy did not develop in our Alport CKD model by echocardiography or heart weight.^36^ Other investigators have shown cardiac hypertrophy in Alport mice on a background different than the C57Bl6J background of our model.^38^ In the present studies, heart weights were not increased by CKD (Supplemental Figure 2).

### Vascular (Aortic) Ca and Gene Expression in Alport Mice

**Figure 6:**
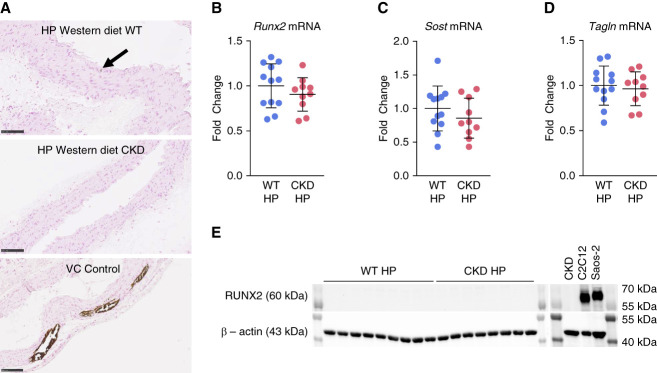
**VC is absent in Alport CKD model with mice fed Western HP diet.** (A) Histologic images of aortas with Von Kossa stain. Both WT and CKD animals showed no significant evidence of VC (*n*=10) as compared with aortic tissue (VC Control) from a mouse model of VC (DBA/2J mouse with CKD and VC induced by 6 weeks of 0.2% adenine followed by 6 weeks of 0.2% adenine and 0.9% high-phosphate diet). There was a presence of approximately 0–3 areas of punctate, <10 micron points of positive stain in full length of axial aorta in both WT and CKD mice (black arrow). Bar is 100 microns. (B–D). Aortic mRNA from whole tissue lysates of WT and CKD mice fed high-phosphate diet (*n*=8–9). *Runx2*, a marker of osteoblastic transition (B), *Sost* (sclerostin), an osteocytic marker (C), and *Tagln* (transgelin, or sm22a), a marker of VSMC differentiation (D), all show no significant change of overall expression between groups using ANOVA with Tukey multiple comparison test. All target genes are displayed normalized to *Rplp13*. Data are represented as means±SD. (E) Western blot showing no RUNX2 protein expression in whole aorta lysates of WT and CKD mice on high-phosphate diet (*n*=8–9). Saos-2 and C2C12 osteoblastic cell lines are shown as positive controls for RUNX2. Saos-2 is loaded in equivalent mass to the samples. *β*-actin (multiplexed with RUNX2) was used as a housekeeping protein for normalization. (A–E) Includes data limited by animals with associated BUN <35 for WT and >43 for CKD. See Supplemental Figure 4 for multiplexed color blot. *Runx2*, runt-related transcription factor 2; VC, vascular calcification; VSMC, vascular smooth muscle cell.

VC as detected by von Kossa staining of aortic sections from 190-day-old Alport CKD mice fed the Western high-Pi (HP) diet was absent (Figure 6A). Aortic *Runx2* mRNA levels (the key marker of VSMC transdifferentiation to osteoblastic phenotype) were not increased (Figure 6B) nor were aortic *Sost* levels (Figure 6C). Furthermore, aortic *Tagln* (sm22*α*) mRNA levels (a marker of VSMC differentiation) were not decreased (Figure 6D). Runx2 protein levels, as detected by Western analysis, were undetectable in aortas from Western high Pi-fed WT and Alport CKD mice (Figure 6E).

## Discussion

Our results report the new discovery that a switch from a vegetable protein-based diet to an animal-based protein HP diet decreases components of cardiac OXPHOS in the presence of normal renal function. Furthermore, other elements of the CKD-MBD were affected by the Western style high-Pi diet in mice with normal renal function. Renal membrane Klotho levels were reduced, and FGF23 and PTH were significantly increased within their normal ranges. Therefore, the diet change prepared the mice for the stimulus of CKD or, in other words, primed them. The effects of the Western-type HP diet on increasing PTH and FGF23 levels in WT mice probably represent adaptations to maintain phosphate homeostasis and normal blood phosphorus levels. However, the reduction in renal klotho levels is potentially even more important. Reductions in klotho induce resistance to FGF23 signaling.^46^ By inducing FGF23 resistance, the mice were especially primed for the cardiac effects of CKD. In CKD, pathologic cardiac FGF23 signaling is through the fibroblast growth factor receptor type 4; this does not happen in the presence of klotho, which directs FGF23 to the FGFR1c and 3. In this study, we used a model of Alport CKD that has been previously shown to develop no significant changes in heart function by echocardiography and no increases in heart rate or BP.^36^ Placing these mice on a Western HP diet does not induce VC or cardiac hypertrophy. Therefore, the resultant cardiac mitochondrial dysfunction is an early disease stage, one that will eventually lead to relevant myocardial injury as suggested by Fuchs *et al*.^38^

The data reported here show that changing from a vegetable protein-based diet to an animal protein HP Western-type diet worsens the severity of the CKD-MBD. On the Western-type HP diet, Alport CKD mice showed increased blood phosphorus, PTH, and FGF23 levels, decreased blood calcium, and decreased kidney klotho levels compared with their WT littermates. The increases in blood phosphorus, the hypocalcemia, and FGF23 levels in CKD were worse with mice on the Western-type HP diet at 190 days old than on the vegetable protein-based regular laboratory chow at 225 days old. Since phosphorus,^47^ FGF23,^48^ CKD,^49,50^ and klotho^51,52^ are all factors contributing to aging, it is possible that they all work in aging through the functions of klotho.^53^ Since klotho reductions are among the earliest perturbations in the development of the CKD-MBD,^54^ it looms as an attractive target for intervention in the CKD-MBD syndrome.

These results are consistent with previous studies, including a recent feeding study of phosphorus additives.^55–57^ They accentuate the key role of phosphate in our animal-based protein Western diet. This suggests that avoiding processed foods with phosphate-based preservatives (which are similar to the Pi addition in our diet) and substituting vegetable protein for meats when possible would provide efficacy to the treatment of CKD and the CKD-MBD.^58,59^ Thus, our results contribute to the developing field of precision nutrition in CKD.^60^ Furthermore, given that sodium-glucose transport 2 inhibitors worsen CKD-MBD,^61–63^ including increase of serum phosphate and FGF23, the results herein open the opportunity to test whether diet modification improves efficacy of sodium-glucose transport 2 agents in slowing progression of CKD. We show that the Western-type HP diet affected renal klotho levels independent of kidney function. Since phosphate, FGF23, PTH, CKD, and klotho have been suggested to be premature aging-associated factors, we hypothesize that klotho deficiency could be the unifying pathophysiologic mechanism tying all of the factors together.^64^ Our klotho data suggest that the aging-like effects of CKD may be mediated through our diet, that the fracture prevalence of CKD may relate to dietary effects on PTH levels, and that the CV effects of CKD may relate to dietary effects on FGF23 and klotho.^65^

We previously reported the development of an Alport CKD model without VC, vascular stiffness, or hypertension.^36^ In this study, the addition of a Western-type HP diet to this model did not promote smooth muscle transdifferentiation and VC. This is despite the observed hyperphosphatemia, and the role of phosphate to affect the Na-Pi cotransporter Pit-1 in renal proximal tubules and VSMC.^66^ The resistance of the C57Bl/6J strain background to VC was not overcome. This provides an opportunity to study the progression of CKD-MBD pathophysiology without the contribution of VC. We have used the opportunity to show that kidney disease and the CKD-MBD directly affect cardiac metabolism independent of vascular disease and extend these results herein.^36^

There are limitations in the data presented here. Measurement of soluble klotho has been fraught with lack of adequate validation of the multiple ELISA assays available. For instance, the most widely used ELISA (IBL International, Japan) was not validated during an analysis of klotho in the Chronic Renal Insufficiency Cohort studies.^67^ This calls into question the detection of purported klotho proteins by the ELISAs in global klotho knockout mice and contributes to the lack of comparability between various assays.^68^ At the time of this manuscript preparation, we did not have sufficient serum to readdress the issue and independently validate the ELISAs currently used. Second, we did not investigate calciprotein particles in our mice with absence of conduit arterial disease to see if these were characteristic of noncalcifying particles. Furthermore, because we had already reported that the mice without conduit arterial disease had a milder renal osteodystrophy,^36^ not harboring the abnormal osteoblastic activity that is an important component of excessive PTH activity and CKD osteodystrophy,^69^ we deferred on assessing the osteodystrophy of the cohorts reported herein. Thus, the bone-vascular paradox was not interrogated. Despite the robust changes in FGF23 that we observed, we did not assess anemia and iron deficiency as contributing to this aspect of the CKD-MBD.^70^

The two diets used in the studies differed in more than just Pi levels or protein sources. Thus, we cannot attribute the changes we observed specifically to Pi or to the protein source. Furthermore, the difference between the two diets in calcium content (0.8% in the vegetable protein-based diet and 0.6% in the animal-based HP diet) with the absence of calcitriol measurements could confound the interpretation of PTH, FGF23, and klotho levels in our studies. The differences in diet mineral composition make phosphate-specific conclusions provisional as they affect levels of Ca-PO_4_ complexes, ionized Ca, and ionic Pi.

Because of the variability of CKD severity in Alport models, including ours, we chose to remove Alport mice without significant CKD and WT mice with evidence of reduced renal function from the analyses. The objective was to use the model to study the CKD-MBD stimulated by diet in mice with Alport CKD and WT controls. Eliminating mice that did not qualify was eliminating a bias against our objective. A limitation is that exclusion of study animals by BUN values may mask direct effects of Western HP diet on CKD progression. Further, although BUN is highly correlated to kidney fibrosis in this model and cost effective to measure, it is not a direct GFR measurement. Another consideration is the reduced euthanasia date of the Western HP WT and CKD animals to preserve animals in the CKD group. This may have had an effect of decreased BUN and fibrosis in those mice due to less advanced Alport CKD. If so, the contribution of CKD-MBD in those animals that is not stimulated by the Western high-phosphate diet may be less developed. It could be speculated that addition of Western HP diet to 225 day old animals would have further increased serum phosphate, PTH, and FGF23 and lowered tissue klotho or cardiac mitochondrial function in the CKD HP group. The difference in ages of 35 days between WT animals in the two diet groups we expect is unlikely show effect in the CKD-MBD or mitochondrial function, but the additional variable complicates interpretation.

In summary, our data provide strong preclinical evidence to support the dietary approach to CKD management. While Pi restriction is not a new concept, relatively simple real-life changes to diet, such as avoiding phosphate-based food preservatives and increasing vegetable protein intake while decreasing meat intake, could be more strongly stressed. This could potentially provide efficacy in delaying progression of CKD-associated cardiac pathology in individuals with modifiable high Pi intake. Clinical trials are needed to demonstrate this.

## Supplementary Material

## Data Availability

Original data generated for the study are or will be made available in a public access repository upon publication. Data Type: Published Material. The data that support the findings of this study are openly available in Washington University School of Medicine's Institutional Repository—Digital Commons Data@Becker, at http://doi.org/10.17632/mv3s66nkmb.1, reference number DOI: 10.17632/mv3s66nkmb.1.

## Declarative Statements

All animal experiments were conducted in accordance with the NIH Guide for the Care and Use of Laboratory Animals or an equivalent standard that meets or exceeds the ethical and welfare requirements outlined in the NIH Guide. All protocols were approved by the appropriate institutional animal care and use committee.

## Supplemental Material

Supplemental Figure 1. Plasma c-terminal FGF23 ELISA shows highest protein levels with Alport CKD mice on HP Western diet.

Supplemental Figure 2. Western high-phosphate diet does not induce hypertrophy in Alport CKD mice.

Supplemental Figure 3. Uncut blots.

Supplemental Figure 4. Multiplexed color blot.
