# Supplementary material for: A Western Diet High in Phosphate Primes the Development of the CKD-Mineral Bone Disorder in an Alport Syndrome Model
Source: Kidney360. 2026 Jan 2;7(5):955–68. doi: 10.34067/KID.0000001065 (PMC13229436; doi:10.34067/KID.0000001065)
Supplement: Supplementary file 1 [file kidney360-7-0955-s001.pdf]

ASN American Society of Nephrology | JASN Journal of the American Society of Nephrology | KIDNEY360 Accessing Our World From Every Angle | CJASN Clinical Journal of the American Society of Nephrology

ASN Journal Disclosure Form

As per ASN journal policy, I have disclosed any financial relationships or commitments I have held in the past 36 months as included below. I have listed my Current Employer below to indicate there is a relationship requiring disclosure. If no relationship exists, my Current Employer is not listed.

B. Finck reports the following:
Employer: Washington University; and Patents or Royalties: Washington University.

I understand that the information above will be published within the journal article, if accepted, and that failure to comply and/or to accurately and completely report the potential financial conflicts of interest could lead to the following: 1) Prior to publication, article rejection, or 2) Post-publication, sanctions ranging from, but not limited to, issuing a correction, reporting the inaccurate information to the authors' institution, banning authors from submitting work to ASN journals for varying lengths of time, and/or retraction of the published work.

Name: Brian Finck
Manuscript ID: K360-2025-000856R1
Manuscript Title: A Western Diet High in Phosphate Primes the Development of the CKD-MBD in an Alport Syndrome Model
Date of Completion: October 14, 2025
Disclosure Updated Date: October 14, 2025

ASN Journal Disclosure Form

As per ASN journal policy, I have disclosed any financial relationships or commitments I have held in the past 36 months as included below. I have listed my Current Employer below to indicate there is a relationship requiring disclosure. If no relationship exists, my Current Employer is not listed.

C. Halling reports the following:
Employer: Washington University St Louis

I understand that the information above will be published within the journal article, if accepted, and that failure to comply and/or to accurately and completely report the potential financial conflicts of interest could lead to the following: 1) Prior to publication, article rejection, or 2) Post-publication, sanctions ranging from, but not limited to, issuing a correction, reporting the inaccurate information to the authors' institution, banning authors from submitting work to ASN journals for varying lengths of time, and/or retraction of the published work.

Name: Carley Halling
Manuscript ID: K360-2025-000856R1
Manuscript Title: "A Western Diet High in Phosphate Primes the Development of the CKD-MBD in an Alport Syndrome Model"
Date of Completion: October 14, 2025
Disclosure Updated Date: October 14, 2025

ASN American Society of Nephrology JASN Journal of the American Society of Nephrology KIDNEY360 Accessing Our World From Every Angle CJASN Clinical Journal of the American Society of Nephrology

ASN Journal Disclosure Form

As per ASN journal policy, I have disclosed any financial relationships or commitments I have held in the past 36 months as included below. I have listed my Current Employer below to indicate there is a relationship requiring disclosure. If no relationship exists, my Current Employer is not listed.

K. Hruska reports the following:
Employer: Washington University in St. Louis

I understand that the information above will be published within the journal article, if accepted, and that failure to comply and/or to accurately and completely report the potential financial conflicts of interest could lead to the following: 1) Prior to publication, article rejection, or 2) Post-publication, sanctions ranging from, but not limited to, issuing a correction, reporting the inaccurate information to the authors' institution, banning authors from submitting work to ASN journals for varying lengths of time, and/or retraction of the published work.

Name: Keith A. Hruska
Manuscript ID: K360-2025-000856R1
Manuscript Title: A Western Diet High in Phosphate Primes the Development of the CKD-MBD in an Alport Syndrome Model
Date of Completion: October 20, 2025
Disclosure Updated Date: October 20, 2025

ASN American Society of Nephrology JASN Journal of the American Society of Nephrology KIDNEY360 Accessing Our World From Every Angle CJASN Clinical Journal of the American Society of Nephrology

ASN Journal Disclosure Form

As per ASN journal policy, I have disclosed any financial relationships or commitments I have held in the past 36 months as included below. I have listed my Current Employer below to indicate there is a relationship requiring disclosure. If no relationship exists, my Current Employer is not listed.

H. Patel has nothing to disclose.

I understand that the information above will be published within the journal article, if accepted, and that failure to comply and/or to accurately and completely report the potential financial conflicts of interest could lead to the following: 1) Prior to publication, article rejection, or 2) Post-publication, sanctions ranging from, but not limited to, issuing a correction, reporting the inaccurate information to the authors' institution, banning authors from submitting work to ASN journals for varying lengths of time, and/or retraction of the published work.

Name: Hiral Patel
Manuscript ID: K360-2025-000856R1
Manuscript Title: A Western Diet High in Phosphate Primes the Development of the CKD-MBD in an Alport Syndrome Model
Date of Completion: October 16, 2025
Disclosure Updated Date: October 16, 2025

ASN American Society of Nephrology | JASN Journal of the American Society of Nephrology | KIDNEY360 Accessing Our World From Every Angle | CJASN Clinical Journal of the American Society of Nephrology

ASN Journal Disclosure Form

As per ASN journal policy, I have disclosed any financial relationships or commitments I have held in the past 36 months as included below. I have listed my Current Employer below to indicate there is a relationship requiring disclosure. If no relationship exists, my Current Employer is not listed.

M. Williams reports the following:
Ownership Interest: QCLN, FEMS, RDVY, IHI, PTNQ, COWZ, DBEF, IVV, ICSH, SCHD, VTI, AVUV, BX, FTXN, ABBV, AMZN, HD, MA, NVDA, QJUN, MLPX, XMMO, HYG, CALF, XLK, ANGL, VWO, VTI, VTV

I understand that the information above will be published within the journal article, if accepted, and that failure to comply and/or to accurately and completely report the potential financial conflicts of interest could lead to the following: 1) Prior to publication, article rejection, or 2) Post-publication, sanctions ranging from, but not limited to, issuing a correction, reporting the inaccurate information to the authors' institution, banning authors from submitting work to ASN journals for varying lengths of time, and/or retraction of the published work.

Name: Matthew James Williams
Manuscript ID: K360-2025-000856R1
Manuscript Title: A Western Diet High in Phosphate Primes the Development of the CKD-MBD in an Alport Syndrome Model
Date of Completion: October 16, 2025
Disclosure Updated Date: October 16, 2025
