# Supplementary material for: A Western Diet High in Phosphate Primes the Development of the CKD-Mineral Bone Disorder in an Alport Syndrome Model
Source: Kidney360. 2026 Jan 2;7(5):955–68. doi: 10.34067/KID.0000001065 (PMC13229436; doi:10.34067/KID.0000001065)
Supplement: Supplementary file 2 [file kidney360-7-0955-s002.pdf]

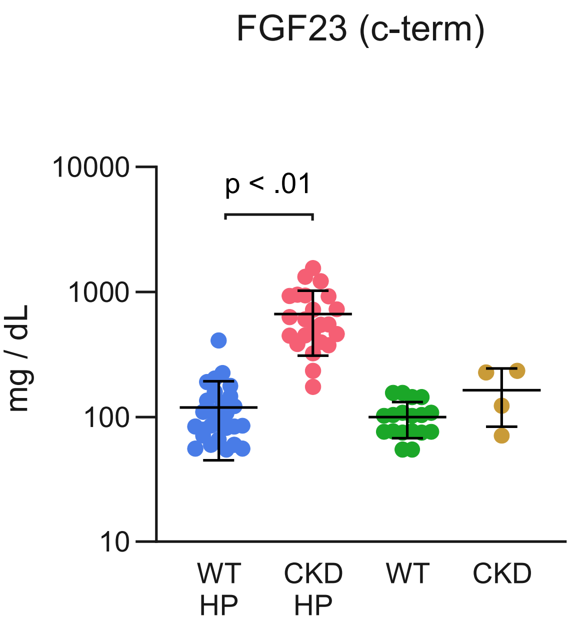

Supplemental Figure 1. Plasma c-terminal FGF23 ELISA shows highest protein levels with Alport CKD mice on high-phosphate (HP) Western diet. Groups include WT and Alport CKD mice fed high-phosphate Western diet or vegetable-based diet. C-terminal FGF23 values likely read lower than Intact FGF23 values (**Figure 3**) due to increased number of freeze-thaws in samples. Significance differences between groups was determined using Kruskal-Wallis H test with Bonferroni correction for multiple pairwise comparisons. Western diet: WT, n = 28; CKD, n = 24. Vegetable diet WT, n = 9; CKD, n = 4. Includes data limited by animals with associated BUN <35 for WT and >43 for CKD. Data are represented as means ± SD and are displayed on log10 scale.

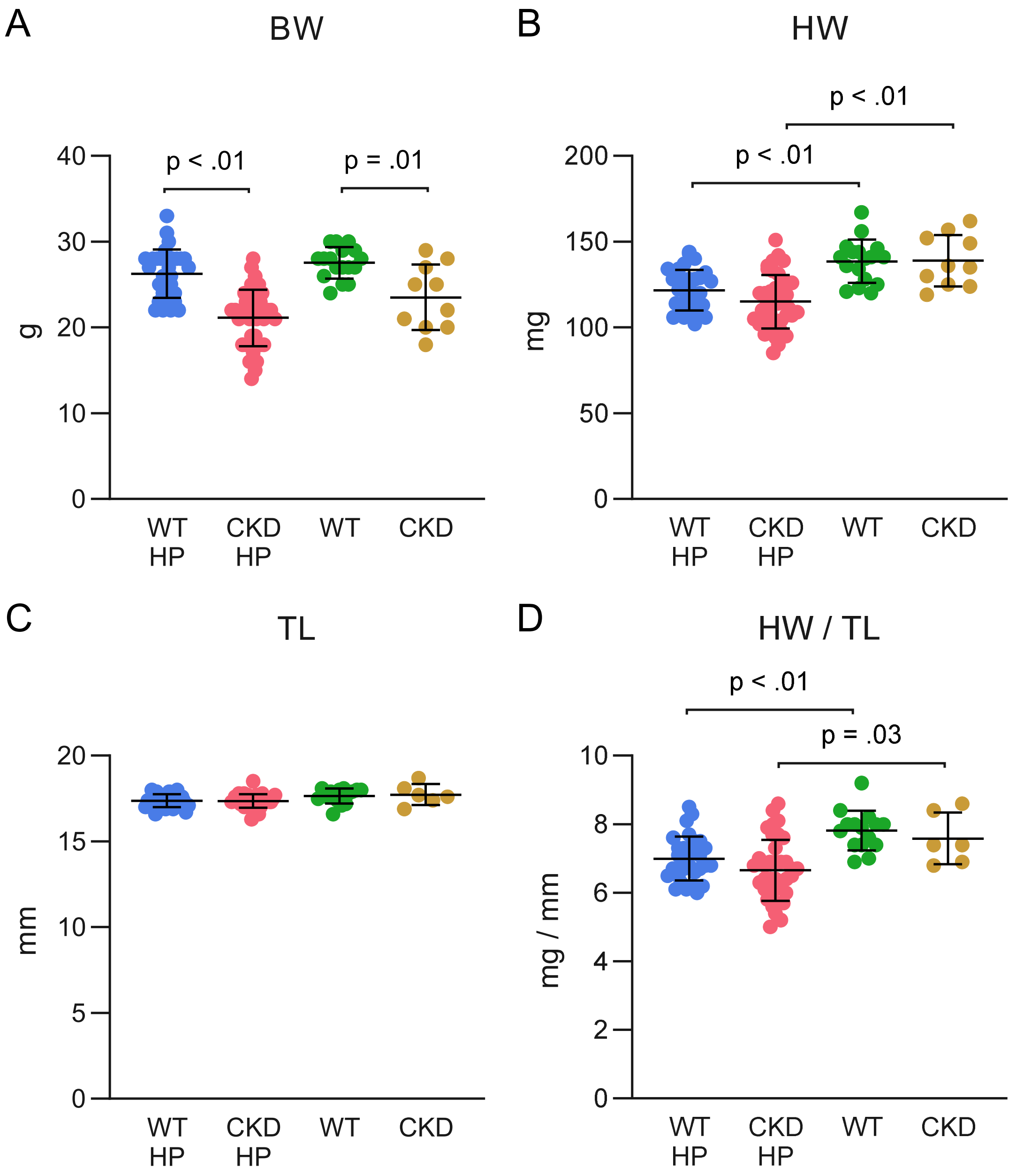

Supplemental Figure 2. Western high-phosphate diet does not induce hypertrophy in Alport CKD mice. Groups include WT and Alport CKD mice fed high-phosphate Western diet (HP) or vegetable-based diet. **A**: Alport CKD induces a cachexia in mice fed either diet. **B**: Heart weight is not changed with CKD compared to wild type littermates in either diet group, however, heart weights are reduced with addition of Western high-phosphate diet in WT or Alport CKD mice. **C**: Tibia length is not significantly changed across the groups. Tibia length is used to normalize heart weight measurements and account for differences in body size independent of body weight fluctuations caused by CKD. **D**: Heart weight normalized to tibia length data supports the group trends shown in **B**. Significant differences between groups were determined using ANOVA with Tukey's multiple comparison test. Western diet: WT, n = 31; CKD, n = 38-41. Vegetable diet: WT, n = 15-17; CKD, n = 6-10. Includes data limited by animals with associated BUN <35 for WT and >43 for CKD. Data are represented as means ± SD.

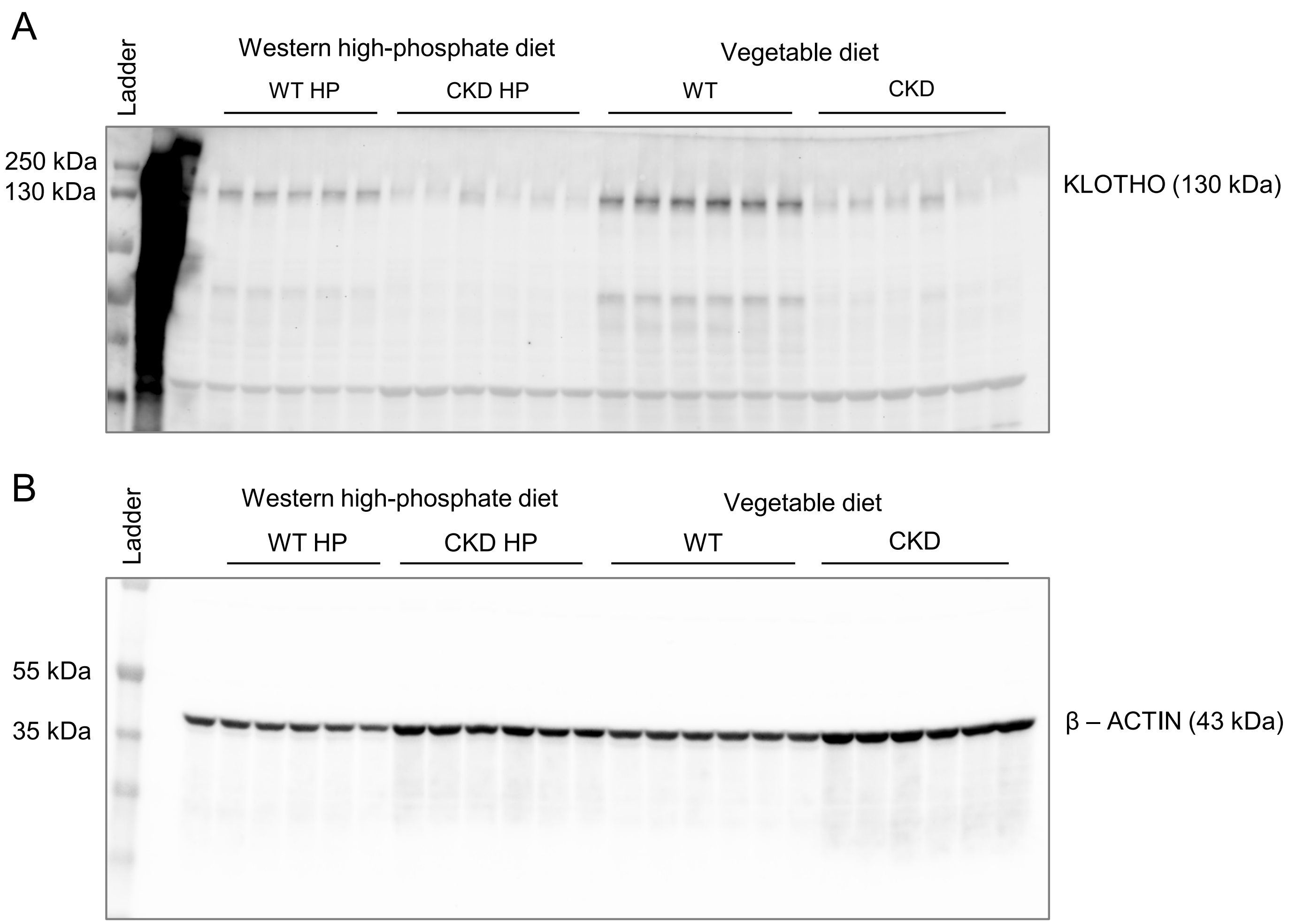

Supplemental Figure 3. Expanded, uncut immunoblots from Figure 4A. **A:** Klotho. **B:** Beta-actin.

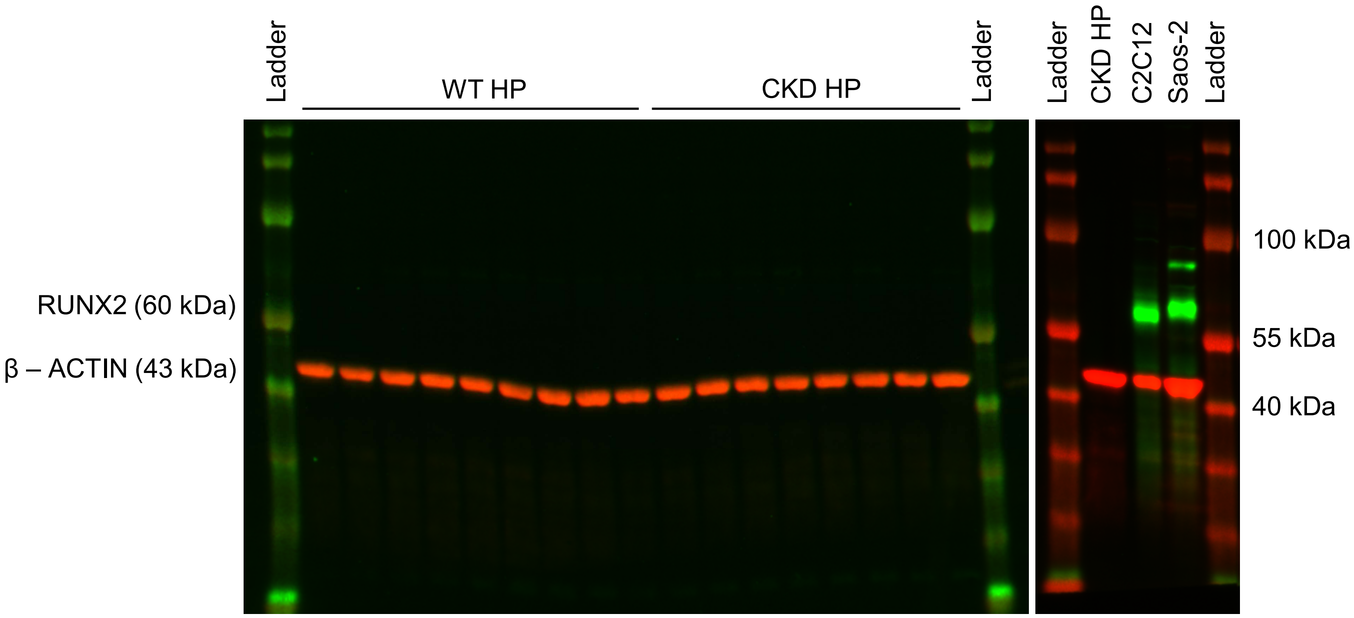

Supplemental Figure 4. Expanded, multiplexed immunoblots from Figure 6E. Green channel is RUNX2, and red channel is Beta-actin.
